# Preferences and decisional considerations relating to opioid agonist therapy among Ukrainian people who use drugs: A conjoint analysis survey

**DOI:** 10.1371/journal.pgph.0002725

**Published:** 2024-01-26

**Authors:** Daniel J. Bromberg, Lynn M. Madden, Liana Fraenkel, Dharushana Muthulingam, Delaney Rhoades, Sergii Dvoriak, Kostyantyn Dumchev, Iryna Pykalo, Frederick L. Altice

**Affiliations:** 1 Department of Social and Behavioral Sciences, Yale School of Public Health, New Haven, Connecticut, United States of America; 2 APT Foundation, New Haven, Connecticut, United States of America; 3 Division of Infectious Diseases, Yale School of Medicine, New Haven, Connecticut, United States of America; 4 Division of Infectious Diseases, Washington University School of Medicine, St. Louis, Missouri, United States of America; 5 European Institute of Public Health Policy, Kyiv, Ukraine; 6 Department of Epidemiology of Microbial Diseases, Yale School of Public Health, New Haven, Connecticut, United States of America; University of Alberta, CANADA

## Abstract

Scaling up opioid agonist therapies (OAT) is the most effective strategy to control combined HIV and opioid epidemics, especially in Eastern Europe and Central Asia (EECA), where HIV incidence and mortality continue to increase. Patient concerns about OAT, however, have undermined scale-up. The objective of this study is to understand Ukrainian opioid use disorder patient preferences about OAT to guide the development of an evidence-informed decision aid for clinical decision-making. We conducted a conjoint-based choice (CBC) survey. Participants were asked to about their preferences relating to 7 attributes of OAT (cost, dosing frequency, concerns about withdrawal symptoms, adverse side effects, improvements in quality of life, precipitation of withdrawal and legislative requirements to be registered as a drug dependent person) and 20 attribute levels for receiving OAT under differing potential treatment constraints. Data were analyzed using Hierarchical Bayesian models. Using respondent-driven sampling and random sampling, we recruited 2,028 people who inject drugs with opioid use disorder. Relative importance (RIS) and partial-worth utility scores (PWUS) were used to assess preferences for attributes and thresholds within each attribute. Cost and dosing frequency were the most important attributes (RIS = 39.2% and RIS = 25.2%, respectively) to potential patients, followed by concerns about withdrawal symptoms (RIS = 10.8%), adverse side effects (RIS = 9.0%), quality-of-life improvement (RIS = 7.5%), precipitation of euphoria (5.2%) and requirement to be registered as a drug- dependent person (RIS = 3.1%). The monthly cost-threshold for willingness-to-pay was 1,900 UAH ($70 USD). In Ukraine, where both governmental and private OAT clinics have emerged and provide markedly different delivery strategies, preferences are mostly driven by out-of-pocket expenses, despite many patients being willing to pay for OAT. Programmatic demands (flexibility and ease of acquiring medications) remain an important consideration while for a minority, clinical concerns about withdrawal symptoms, adverse side effects and OAT impact on life play a smaller role.

## Introduction

Eastern Europe and Central Asia (EECA) has the fastest growing HIV epidemic in the world [1]. HIV incidence in this region is concentrated among people who inject drugs (PWID) and is driven primarily by suboptimal HIV treatment and prevention in this group and their sexual partners [1]. The country of Ukraine, specifically, has the highest HIV prevalence in Europe (1.2%) driven by high rates of drug injection using non-sterile equipment [2].

Maintenance with opioid agonist therapies (OAT), including methadone or buprenorphine, is the gold standard treatment for OUD [3] and, when properly scaled-to-need, can control HIV and opioid epidemics. For example, mathematical modeling from Ukraine [4] showed that OAT scale-up from 3% (the current coverage) to 20% coverage of PWID in the country would be the most effective intervention to reduce new HIV infections, and would also be the most cost-effective strategy for doing so [5, 6]; however, despite the urgency of scaling up OAT, multiple barriers remain. One large study of 1,614 PWID explored 24 previously identified barriers to OAT [7–15] found logistical constraints (i.e., requirements for treatment entry and continuity) and perceptions about OAT (i.e., treatment efficacy, safety, tolerability or inability to discontinue) to be the two most important barriers to OAT entry [16]. These logistical constraints have been the focus of recent Ukrainian policy.

The opioid agonist therapy (OAT) environment in Ukraine has undergone significant changes in recent years. Until 2016, OAT in the form of methadone was exclusively provided at governmental clinics. The administration of methadone was primarily conducted as a daily, directly supervised therapy; however, due to the onset of the COVID-19 pandemic and the ongoing war in Ukraine, there has been a notable expansion in the availability of take-home dosing for methadone [17, 18]. This change has allowed individuals to have more flexibility in managing their treatment. While buprenorphine is also available as an OAT option in Ukraine, it is rarely prescribed, and as a result, methadone remains the predominant choice for OAT in the country.

In late 2016, Ukrainian legislation overseeing OAT delivery (Order 200) [14] was amended to allow treatment outside specialty addiction clinics (e.g., private clinics) and allowed patients with documented sobriety for six consecutive months to receive up to 10 days of take-home dosing (THD). In 2020, Emergency guidance in response to the COVID-19 pandemic further allowed more flexible THD for anyone deemed clinically stable irrespective of time on OAT [19]. Another development in the OAT landscape occurred in 2021 with the introduction of methadone in Ukrainian prisons, aiming to address the needs of incarcerated individuals struggling with opioid addiction. More relaxation of THD during the 2022 invasion of Ukraine by Russia also occurred [20], and further accelerated the adaptation of new models of OAT delivery [17, 18, 20, 21]. As OAT delivery continues to be in flux, understanding patient preferences can inform emerging models of care and scale-up. Preferences can guide the development of implementation tools like informed decision aids, to provide a patient-centered strategy to improve individual and public health [22–24].

Decision aids are evidence-informed tools that present risks and benefits, guiding patients in selecting treatment options aligned with their preferences. The development of such tools is especially crucial in Ukraine where numerous barriers have undermined OAT scale-up targets and treatment options are evolving. Decision aids provide accurate information about the effectiveness of available treatments, including potential attributes [25–27], and focus on patient-centered health outcomes [28, 29]. As individuals navigate medication options, they also develop ownership in the treatment plan, potentially increasing engagement in care.

To inform the development of a decision aid, we used a choice-based conjoint (CBC) experiment to identify patient preferences and priorities.

## Methods

### Ethics statement

This study was granted ethical approval by the Institutional Review Boards at Yale University (2000021361) and the Ukrainian Institute for Public Health Policy, both in 2019.

CBC analysis originates in marketing research where it is used to measure client utility tradeoffs for differing attributes of a given good or service [30]. It has also been successfully used in health research [31], assessing patients’ trade-offs in risks and benefits among potential options. In CBC, patients choose between profiles composed of differing attributes, with trade-offs revealing what they value.

Between July 2020 and May 2021, we conducted a randomly sampled, cross-sectional survey that included PWID with OUD who were on OAT, previously on OAT and never on OAT; a CBC survey was included along with other items. Trained Ukrainian research assistants administered the survey through a computer-assisted survey instrument (CASI) in private rooms in five cities of Ukraine (Dnipro, Kyiv, L’viv, Mykolaiv, Odesa).

### Eligibility and recruitment

Eligibility criteria included: 1) having ever injected opioids and meeting ICD-10 criteria for opioid dependence; 2) age 18 years or older; 3) living or working in one of five target cities; and 4) ability to provide informed consent and complete the survey. Recruitment methods have been previously described [32–34]. For those currently or previously on OAT, they were randomly selected from a list generated for each city; the number selected was based on the target sample sizes. For those not on OAT, respondent-driven sampling (RDS) was used where primary respondents (called “seeds”; see S2 Table) were identified based on sex, having large network sizes (i.e., knowing other PWID in their city) and age, in various parts of each city. Participants recruited using RDS were allocated to the OAT groups if they stated prior or current OAT involvement, which was verified in the national OAT registry.

### Survey design

Survey development was informed by in-depth, client-centered focus groups with a collective 199 PWID currently and previously enrolled in OAT, as well as those who were treatment-naïve and published elsewhere [11–15]. These findings, including barrier severity [16], patients’ willingness to enroll [34], and willingness to pay along with cost thresholds [33], informed the attributes and levels in the CBC component of the survey, including 7 attributes, with up to 4 levels per attribute, reflecting patient priorities in treatment consideration. Additional interviews were conducted to guide further demarcation of attribute levels, including the terminology used by potential patients. The attributes and levels are included in **S1 Table**. The survey was developed in English, translated to Russian, then back translated to English to ensure integrity of translation [35].

The survey was designed in Sawtooth version 9.2.0. We used a complete enumeration strategy to ensure that choice sets were constructed randomly. Every participant completed 12 random choice sets. None of the tasks were fixed. Each attribute included the option to not choose a medication (and either choose detoxification at a government clinic or to continue illicit drug use). Before conducting the survey, patients watched a brief video defining terms.

### Procedures

Participants were randomly recruited. Individuals currently or previously on opioid agonist treatment (OAT) were chosen randomly from the Ukrainian national registry called SyRex. Participants who had never received OAT were recruited using respondent-driven sampling (RDS).

In the case of respondent-driven sampling (RDS), initial participants or "seeds" were purposefully selected based on their gender, location, and age. Subsequently, these participants could refer up to three peers to participate. Inclusion criteria for the RDS sample included self-reported recent drug injection and not being on OAT. To ensure proper enrollment of participants, whether through random sampling or RDS, each participant was provided with a coupon containing the study location, contact details of the research assistant, and a unique study ID. The format of the coupons varied for participants randomly selected and those recruited through RDS. The RDS coupons were specially coded to link recruiters with recruits and consisted of two detachable parts.

### Analyses

Conjoint analysis includes all responses to the choice tasks to estimate the average utilities for the whole sample. Hierarchical Bayes (HB) in Sawtooth Software then uses the average to estimate the *part-worth utility scores* (PWUS) for each level, for each individual respondent. This was performed using a Markov Chain Monte Carlo simulation approach, including 10,000 simulations to satisfy convergence assumptions.

PWUS reflect the relative preferences for levels within a given attribute. In other words, they are the numeric representation of the relative importance that respondents give to an attribute on average. PWUS are interval values centered at zero,. The level with the highest PWUS is most preferred, and the level with the lowest (generally, most negative) score is least preferred. The *relative importance score* (RIS) for each attribute is calculated by dividing part-worth utility by the sum of all utility scores and multiplying by 100. Greater RIS signify more importance for a given attribute.

Latent class analysis was used to identify clusters of patients according to their preferences regarding OAT; the Bayesian Information Criterion (BIC) and sample size adjusted BIC (aBIC) were used to determine the number of latent classes of best fit. BIC and aBIC both compare the relative fit of models, and are widely used in latent class analyses. Minimizing BIC and aBIC determines the number of latent classes that best fit the data in a latent class analysis [36].

## Results

All 2028 recruited participants were included in the analysis. Over three-quarters (78%) of the sample was male, the average age was 40 years (SD: 8 years), and 29.9% had previously served sentences in prison (not including pre-trial detention).

**Table 1** summarizes the RIS of the seven attributes. Cost (“How much does it cost?”) was the most important attribute (39.2% of all relative importance), followed by frequency of dosing (RIS = 25.2%). The two attributes with the lowest RIS were governmental registration (RIS = 3.1%) and whether the substance precipitated euphoria (RIS = 5.2%). Part-worth utility scores for each level within the seven attributes are displayed in **Table 2.**

**Table 1:** Relative importance scores (%) of OAT program attributes by group.

Attributes	Total	Group 1	Group 2	Group 3	Group 4	Group 5
		*n* = 218	*n* = 375	*n* = 400	*n* = 758	*n* = 374
		(10.3%)	(17.7%)	(18.9%)	(35.8%)	(17.2%)
*Treatment Costs per Month*	39.2	72.5	38.7	36.3	38.7	24.6
*How frequent is dosing*?	25.2	19.8	19.3	35.7	16.9	19.5
*Severity of Withdrawal Symptoms if Treatment Discontinued*	10.8	3.0	4.3	7.7	20.6	15.5
*Adverse Side Effects*	9.0	1.9	18.7	10.1	12.9	15.4
*How much will life get better*?	7.5	0.9	4.4	8.9	1.8	4.7
*Precipitates Euphoria*	5.2	0.8	10.6	1.0	6.3	14.5
*Governmental Registration*	3.1	1.1	4.0	0.4	2.8	5.7

Notes

Relative importance scores reflect the influence that each attribute has on a participant’s decision-making.

**Table 2:** Part-worth utility scores (zero-centered)^*d*^ of OAT program level choices by group.

Attributes and Levels		Group 1 *n* = 218	Group 2 *n* = 375	Group 3 *n* = 400	Group 4 *n* = 758	Group 5 *n* = 374
		(10.3%)	(17.7%)	(18.9%)	(35.8%)	(17.2%)
*Frequency*						
Daily	15.3	10.5	-13.8	15.8	57.6	17.0
Every 10 days	60.5	64.1	74.4	117.1	3.3	59.8
3–4 times/day	-75.9	-74.7	-60.6	-132.9	-60.9	-76.9
*Effects*						
Positive	15.4	-3.1	15.6	31.0	6.3	16.6
Negative	-15.4	3.13	-15.6	-31.0	-6.3	-16.6
*Precipitates Euphoria*						
Yes	-8.7	-2.6	37.0	3.5	22.2	51.1
No	8.7	2.6	-37.0	-3.5	-22.2	-51.1
*Registration*						
Required	-3.2	-4.0	-14.1	-1.4	-9.8	-20.0
Not required	3.2	4.0	14.1	1.4	9.8	20.0
*Cost per month*						
150 UAH ($6 USD)	134.1	270.2	108.3	146.3	171.1	103.7
1900 UAH ($70 USD)	44.7	102.2	117.4	55.4	24.7	28.2
7700 UAH ($285 USD)	-85.3	-135.3	-71.9	-107.7	-96.0	-63.4
10k – 20k UAH ($370–741 USD)	-93.5	-237.2	-153.7	-94.1	-99.8	-68.4
*Side Effects*						
Mild	9.0	-3.0	6.3	32.7	5.2	36.6
Moderate	17.7	8.2	62.2	5.0	42.4	34.5
Severe	-26.7	-5.2	-68.4	-37.7	-47.6	-71.0
*Withdrawal*		-52.32	60.64	-137.59	-34.36	-4.02
Short term, mild	22.0	3.7	17.1	24.7	79.1	33.4
Short term, severe	3.1	8.5	-12.8	4.5	-65.1	37.5
Long term, severe	-25.0	-12.3	-4.4	-29.1	-14.0	-70.9

Notes

*d*: Zero-centered part-worth utility scores imply the positive or negative magnitude of the preference for the level choice in relation the other level options within the same attribute.

Latent class analysis determined that there were five groupings of patients based on their preferences (need to include table with goodness of fit characteristics). These groupings are presented in **Tables 1 and 2**.

In aggregate, the entire sample preferred less expensive, more take-home dosing (THD), fewer withdrawal symptoms if treatment is discontinued, and fewer adverse side effects. Treatment cost was the largest consideration overall, and for each latent class grouping. Only in group 3 was treatment cost tied with another consideration (in-person dosing frequency).

Group 1, the smallest group (10.3% of the total), overwhelmingly considers price as the main consideration when seeking treatment, with all other considerations virtually negligible. All other groups have a mix of considerations. Like the other latent classes, Group 2 is concerned about cost (38.7%) and dosing frequency (19.3%); however, the participants are relatively more concerned about medication side effects (18.7%) than all other groups (mean: 9.0%). Group 3 valued program-level demands the highest (35.7%) of all groups (mean: 25.2%) as well as improved quality of life (mean: 8.9% vs 7.5%). Group 4 was the largest group (n = 758) and considered potential withdrawal symptoms more highly than other groups (mean: 20.6% vs 10.8%). They were least concerned about frequency of dosing (mean: 16.9% vs 25.2%). Group 5 is the least concerned about costs (mean: 24.6% vs 39.2%). Relative to other groups, Group 5 was more concerned that treatment options may precipitate euphoria (mean: 14.5% vs 5.2%). No group considered the need to register with the government to be a significant barrier to treatment (mean: 3.1%); however, relative to other groups, group 5 did have the greatest concern about registration (5.7%).

## Discussion

The key findings are that preferences are: 1) most driven by out-of-pocket expenses, although many patients are willing to pay for OAT; 2) also driven by programmatic demands (flexibility and ease of acquiring medications); and 3) for all but a small group of patients, less driven by non-financial concerns, such as clinical concerns about withdrawal symptoms, adverse side effects and OAT impact on life. These findings are important and inform potential treatment strategies learned from disruptions from both the COVID-19 pandemic and the war have accelerated the need for innovative strategies to guide OAT scale-up in Ukraine. Findings here also provide implications for elsewhere in EECA and globally, where OAT is even more restricted. Findings from this study of patient preferences provide important insights into service delivery models that can greatly expand OAT scale-up, including ones that evolved during the war and were not available when this study was first conducted [17, 21].

Despite Russia’s war in Ukraine, Ukraine has continued to scale-up OAT [21], but only one region (Kharkiv) has met the scale-up goal of OAT coverage greater than 20%. This was achieved through innovations in service delivery, namely the emergence of a private clinic model that requires patients to pay for OAT and related clinical services. This has managed to attract over five times as many patients as the more tightly regulated government system [17], which is free for patients. The private model removes the requirement of governmental registration, provides OAT the same day as requested and more rapidly transitions patients to THD, thereby reducing program demands on patients (and providers). This suggests that several programmatic factors contribute to patient preferences, as long as out-of-pocket expenses are not prohibitive. Importantly, however, governmental clinics have accelerated their own scale-up, markedly shifting patients more rapidly to THD due to COVID-19 and the war [21].

This sample had considerable heterogeneity, much like PWID in most communities and preferences in the five latent class groupings differed. There were no differences in the proportion of patients willing to pay for OAT across the five groups, with nearly a third willing to do so. While concerns about out-of-pocket expenses were the highest relative to all other considerations overall, monthly treatment costs of 150 UAH ($6 USD) or 1900 UAH ($70 USD) were acceptable or preferred in each group. The 1900 UAH ($70 USD) monthly cost threshold is within reach of many participants surveyed and falls within the range of charges levied by the many emerging private clinics (typically in the regions with higher incomes). Out-of-pocket expenses higher than this, however, had negative utility values and were therefore prohibitive. Thus, it is not surprising that patients in Kharkiv, the second largest city in Ukraine and one with relatively high living standards, scaled up OAT primarily through expansion in private clinics as the costs for OAT were within their acceptable range. Importantly, these private clinics also reduced programmatic demands by providing more rapid transition to THD, removing registration requirements and allowing medication choice—methadone or buprenorphine, with the latter costing more [37]. Of interest, although the governmental clinics remain free and have increasingly reduced program demands on patients during the COVID-19 pandemic and the war, they have not expanded buprenorphine availability. It will be interesting to see if these factors will impact the speed of governmental clinic OAT scale-up.

Ukraine’s OAT program still receives support through international donors. While this support will likely remain as Ukraine struggles with the ongoing war, external support for OAT in Ukraine and elsewhere in EECA is time-limited and may result in treatment delivery convergence and/or public/private partnerships.

Though cross-sectional and qualitative studies in Ukraine [11, 13, 15, 38] found that that stated misperceptions about OAT are drivers of low OAT enrollment, CBC differs in methodology as it identifies “revealed” rather than stated preferences, with participants making trade-offs among available treatment options, in a way that better mimics actual decision-making. In cross-sectional or qualitative studies, barriers may be reported but it is unclear how these barriers are prioritized in clinical decision-making. In sharp contrast to these previous findings, concerns about medication side-effects (a main theme of OAT-related myths) and influence on quality of life did *not* play a prominent role in patient preferences, together accounting for only one-sixth (16.5%) of all patient PWUS. Although we did not directly assess OAT myths as drivers of preference, it is assumed that they make up some smaller proportion of this combined utility as most of the myths elaborated ascribed negative impacts on health as a major impediment to OAT scale-up.

Important in these findings is that patient preferences also do not necessarily align with stated barriers to OAT by stakeholders [14]. For example, in a rank-ordering mixed methods assessment of clinicians, strict regulations around providing OAT did align with patient preferences around frequency of dosing, yet lack of regional autonomy in securing OAT or delivering it were the most reported clinician barrier, which did not figure prominently into patient preferences. This suggests that multi-level factors contribute differently to OAT scale-up [14].

The practical implication from this CBC assessment is that reduction in clinical programmatic demands to access and remain on OAT will likely lead to higher enrollment and retention, thus driving up census, as their preferences are aligned with their treatment. Understandably, patients preferred less frequent clinic visits through THD as such demands interfered with other daily priorities (e.g., employment, childcare, engaging with family, etc.). One of key stated goals for PWID is to increase their engagement in meaningful activities with their families (i.e., social and financial support) and communities (i.e., employment), yet demands of daily supervision often conflict with these goals.

We conducted this study at a time of rapid transition in program-level demands on patients in Ukraine, which continues to evolve. Order 200, the legislation governing OAT provision in Ukraine, has increasingly allowed for THD during public health and war crises [14]. During the COVID pandemic and the Russian invasion, decreased employment and income decreased alongside logistical clinical demands that contributed to out-of-pocket costs, which should be incorporated into patient preference decision-making. It is unclear, however, that more flexible THD policies will remain despite increased OAT scale-up during both crises [17, 21] no increased mortality related to THD during the COVID-19 pandemic [39]. Thus, in order to sustain the benefits of OAT scale-up during the time of crisis, it will be important to incorporate these patient preferences as the crisis stabilizes–both for Ukraine but also as other more restrictive settings target increased scale-up.

Frequency of supervised dosing is the primary demand on patients, driving their preferences. There are, however, other additional clinical demands that persist in Ukraine and elsewhere throughout EECA. For example, a vestige of the Soviet system is the requirement of a formal diagnosis of OUD, which legislatively requires a tribunal of three physicians, one of whom must be a narcologist, to observe and attest to the diagnosis, which must be made during an inpatient admission. While many Ukrainian physicians attempt to ensure the inpatient experience is minimal, this practice is not fully enacted consistently throughout the entire country.

One programmatic demand that was not found to be influential in this survey was governmental registration, in contrast to earlier assessments. In Ukraine, as in other post-Soviet countries of EECA, methadone patients are required to formally register with the government. As part of this registration, patients were banned from receiving a driver’s license or working in certain professions. While these regulations persist on paper, in more recent years, they are seldom enforced at the local level, making these less of a perceived concern.

### Limitations

Our survey was conducted prior to the war, and therefore it is unclear how preferences may have changed in the midst of the conflict. The dynamics of the war and its impact on the population’s needs and preferences could potentially influence the validity of the results here presented. The major changes to OAT provision during war in Ukraine have been described previously [17, 18, 20, 21, 40]. Additionally, although we constructed the conjoint survey using expert opinion and qualitative interviews conducted between 2014 and 2021, these methods are not without their limitations. It is possible that some important attributes that could have influenced preferences were inadvertently omitted. While we sampled from five cities in different regions of the country, it is possible that not all localized circumstances were captured. For example, the city of Kharkiv has higher rates of private clinic enrollment than the nationwide average [17]; however, this was not necessarily the case when the present survey was conducted. Social desirability bias is unlikely in our sample as the survey was anonymous and did not focus on stigmatizing behavior beyond the inclusion criteria (all participants were PWID). Despite these limitations, we have taken the necessary steps to ensure the rigor and validity of our study, and we believe our findings provide valuable insights into the subject matter at hand.

### Conclusion

After the collapse of the Soviet Union in the early 1990s, countries of the EECA have evolved into diverse political, economic, and social trajectories. Yet, this region has experienced similar HIV and OUD epidemics, making strategies that focus on diagnosing and treating OUD with evidence-based treatments a central strategy for HIV prevention. Heroin remains the most injected drug throughout the region, injected by over 80% of PWID. The early policy response to opioid injection in Ukraine and throughout EECA was harsh and punitive. OAT was often introduced late in response to the HIV epidemic or not at all in some countries like Russia and Turkmenistan. It remains as a pilot program without growth for many more EECA countries. Moreover, OAT was not introduced as treatment for OUD, but for HIV prevention, which helped shape similar and often negative beliefs about OAT [41]. The findings here, as well as the development of future decision aids, are likely to also be relevant in nearby EECA countries. Beyond EECA, the findings here can significantly inform patient-centered OAT delivery strategies globally, especially in areas confronting concentrated HIV epidemics among PWID. The key takeaway is the universal importance of providing easy and convenient patient care. Despite previous focus on myths and misperceptions surrounding OAT, this study highlights that concerns on the program side may outweigh patient-side myths about treatment, underscoring the need for a comprehensive and patient-centered approach to OAT delivery.

## Supporting information

S1 Text Inclusivity in global research. (DOCX)

S1 Table Description of attributes. (DOCX)

S2 Table Seed characteristics for RDS sampling. (DOCX)
