# Supplementary material for: Preferences and decisional considerations relating to opioid agonist therapy among Ukrainian people who use drugs: A conjoint analysis survey
Source: PLOS Glob Public Health. 2024 Jan 26;4(1):e0002725. doi: 10.1371/journal.pgph.0002725 (PMC10817130; doi:10.1371/journal.pgph.0002725)
Supplement: S1 Table — (DOCX) [file pgph.0002725.s002.docx]

S1 Table: Description of Attributes

| **How often do you need to go for the drug (narcotic substance)? 25%** *Как часто необходимо ходить за препаратом (наркотическим веществом)?* | |
| --- | --- |
|  | Every morning  *Каждое утро* |
|  | Every 10 days  *Каждые 10 дней* |
|  | 3-4 times a day  *3-4 раза в день* |
| **What are the effects? 7.5%**  *Какие эффекты?* | |
|  | No cravings, family relationships and ability to work are improved  *Нет тяги, улучшаются семейные отношения и способность работать* |
|  | Craving for drugs continues and occurs frequently, constant difficulties with work and family relationships  *Тяга к наркотикам продолжается и возникает часто, постоянные трудности с работой и семейными отношениями* |
| **Taking these substances, will I feel "high" and "kumar"? 5.2%**  *Принимая эти вещества, я буду испытывать “кайф” и “кумар”?* | |
|  | No, your condition remains stable, "normal".  *Нет, ваше состояние остается устойчивым, “нормальным”.* |
|  | Yes, very strong "high" and "kumar"  *Да, очень сильный “кайф” и “кумар”* |
| **Is registration required? 3.1%**  *Требуется ли регистрация?* | |
|  | To start treatment, you must register  *Для начала лечения следует зарегистрироваться* |
|  | No registration required  *Регистрироваться не требуется* |
| **How much does it cost? 39%**  *Сколько это стоит?* | |
|  | 150 hryvnia per month  *150 гривен в месяц* |
|  | 1900 hryvnia per month  *1 900 гривен в месяц* |
|  | 7700 hryvnia per month  *7 700 гривен в месяц* |
|  | 10,000 - 20,000 UAH per month  *10,000 - 20,000 гривен в месяц* |
| **What are the possible side effects? 9%**  *Какие возможны побочные эффекты?* | |
|  | Moderate (may affect the quality of daily life, but can be managed at home)  *Умеренные (могут влиять на качество повседневной жизни, но с ними можно справиться дома)* |
|  | Mild (annoying but can be dealt with easily at home)  *Слабые (беспокоящие, но с ними можно справиться дома)* |
|  | Severe (Serious, life-threatening, unwanted side effects that require medical attention)  *Серьезные, угрожающие жизни, нежелательные побочные эффекты, требующие медицинской помощи* |
| **What happens if I stop treatment without medical supervision? 10.8%**  *Что произойдет, если я прекращу лечение без медицинского наблюдения?* | |
|  | Severe and long-term withdrawal symptoms (4-6 weeks)  *Тяжелые и длительные симптомы синдрома отмены (4-6 недель)* |
|  | Mild and short-term withdrawal symptoms (1-2 weeks)  *Слабые и кратковременные симптомы синдрома отмены (1-2 недели)* |
|  | Severe but short-term withdrawal symptoms (1-2 weeks)  *Тяжелые, но кратковременные симптомы синдрома отмены (1-2 недели)* |
