# Supplementary material for: Preferences and decisional considerations relating to opioid agonist therapy among Ukrainian people who use drugs: A conjoint analysis survey
Source: PLOS Glob Public Health. 2024 Jan 26;4(1):e0002725. doi: 10.1371/journal.pgph.0002725 (PMC10817130; doi:10.1371/journal.pgph.0002725)
Supplement: S2 Table — (DOCX) [file pgph.0002725.s003.docx]

S2 Table: Seed characteristics for RDS sampling

| City | Number of seeds | Seeds characteristics |
| --- | --- | --- |
| Kyiv | 3 | 1 female respondent  1 respondent 18 – 25 years old  1 respondent with history of injection opiate drug use less than 2 years |
| Odesa | 3 | 1 female respondent  1 respondent 18 – 25 years old  1 respondent with history of injection opiate drug use less than 2 years |
| Mykolaiv | 3 | 1 female respondent  1 respondent 18 – 25 years old  1 respondent with history of injection opiate drug use less than 2 years |
| Dnipro | 3 | 1 female respondent  1 respondent 18 – 25 years old  1 respondent with history of injection opiate drug use less than 2 years |
| Lviv | 3 | 1 female respondent  1 respondent 18 – 25 years old  1 respondent with history of injection opiate drug use less than 2 years |
| Total | 15 |  |
